# The effectiveness of parasacral transcutaneous electrical nerve stimulation in the treatment of monosymptomatic enuresis in children and adolescents: a systematic review

**DOI:** 10.1590/S1677-5538.IBJU.2023.0618

**Published:** 2024-03-18

**Authors:** Melissa Faria Dutra, José de Bessa, Emerson Coelho Luiz de Almeida, Eleonora Moreira Lima, Mônica Maria de Almeida Vasconcelos, Flávia Cristina de Carvalho Mrad

**Affiliations:** 1 Universidade Federal de Minas Gerais Faculdade de Medicina Unidade de Nefrologia Pediátrica Belo Horizonte MG Brasil Departamento de Pediatria, Unidade de Nefrologia Pediátrica, Faculdade de Medicina, Universidade Federal de Minas Gerais (UFMG), Belo Horizonte, MG, Brasil;; 2 Universidade Estadual de Feira de Santana Departamento de Urologia Feira de Santana BA Brasil Departamento de Urologia, Universidade Estadual de Feira de Santana (UFSC), Feira de Santana, BA, Brasil

**Keywords:** Systematic Review [Publication Type], Nocturnal Enuresis, Transcutaneous Electric Nerve Stimulation

## Abstract

**Background:**

Parasacral Transcutaneous Electrical Nerve Stimulation (PTENS) is a treatment used in enuresis refractory to first-line treatment. This review aimed to evaluate the effectiveness of PTENS in treating monosymptomatic enuresis (MNE) in children and adolescents.

**Methods:**

The study followed the Preferred Reporting Items for Systematic (PRISMA) guidelines. The search was carried out in the following databases: MEDLINE (via PubMed), Web of Science, SCOPUS, Central Cochrane Library and Physiotherapy Evidence Database (PEDro). The selected studies were randomized clinical trials (RCTs). The "Risk of Bias tool for randomized trials" and the "Risk of Bias VISualization" were used to analyze the risk of bias.

**Results:**

Of the 624 studies selected, four RCTs were eligible. Three included 146 children and adolescents aged between six and 16.3 years and used similar PTENS protocols with a frequency of 10 Hz, pulse duration of 700 µs and 20 minutes three times/week. One study enrolled 52 patients aged seven to 14 years used PTENS at home, with a pulse duration of 200 µs and 20 to 60 minutes twice/day. Risk of bias was observed in three studies due to results’ randomization and measurement. Two studies showed a partial response with a reduction in wet nights, one a complete response in 27% of patients, and one showed no improvement.

**Conclusion:**

PTENS reduces wet nights’ frequency but does not cure them, except in 27% of patients in one study. Limited RCTs and data heterogeneity are limitations.

## INTRODUCTION

Monosymptomatic enuresis (MNE) is a condition defined by the International Children’s Continence Society (ICCS) as isolated intermittent urinary incontinence during sleep in children of five years or above, which occurs once a month for three consecutive months and is not caused by any organic factors. If the child has never achieved urinary continence for more than six months, the condition is defined as primary enuresis, and if a relapse occurs after a dry period of at least six months, it is defined as secondary enuresis. Enuresis is considered infrequent if it occurs less than four times a week and frequent if it occurs four or more times a week (1-4). A recent study showed that 9% of six-year-old children had enuresis, with males being more commonly affected. Of this group, 1.2% were diagnosed with MNE (5).

The exact etiology of enuresis is not yet fully understood. It is considered multifactorial (1,2,6-11). One of the main factors involved in the pathophysiology of enuresis are hereditary factors (1,2, 4-6). Jϕrgensen et al. (12) revealed 12 protein-coding genes, including PRDM13, S1M1 and EDNRB, which are particularly interesting because they are involved in the three pathophysiological mechanisms of enuresis: excessive production of nocturnal urine (nocturnal polyuria) due to the altered circadian cycle of antidiuretic hormone, disturbances in the function of bladder (reduction in bladder capacity and nocturnal detrusor overactivity) (12, 13) and inability to wake up when you need to urinate (impaired arousal) (14, 15).

In order to diagnose MNE correctly, it is important to conduct a systematic clinical history along with a thorough physical examination (1, 2, 4, 6, 16). To screen for other lower urinary tract symptoms (17-20), psychological and behavioral comorbidities (21), sleep disorders (22), and constipation (23), standardized and validated questionnaires should be used. Additionally, a bladder and bowel diary, dry night diary, and a urinalysis (which includes a urine dipstick to detect glycosuria and leukocytosis) should be ordered (1, 2, 6, 16). If children experience polyuria and polydipsia, it is recommended to test for diabetes insipidus (6). If there is suspicion of neurogenic or anatomical problems in the bladder, additional tests such as urodynamic evaluation may be necessary (16).

Treatment options for MNE include urotherapy, enuresis alarm, and medications such as desmopressin acetate (DDAVP), anticholinergics, and tricyclic antidepressants. A combination of these modalities can also be used (1-4, 6). Currently, the first-line treatments for MNE are considered enuresis alarms and DDAVP (4).

The enuresis alarm is a behavioral or conditioning treatment that requires the participation and motivation of both children and their parents (6), Although it has a success rate of 50 to 70%, it also has a high discontinuation rate (24). However, one of its advantages is the low probability of causing adverse effects (1-4, 6).

DDAVP is a synthetic antidiuretic hormone that reduces urine production during the night (25). Although it is generally safe for long-term use, it can cause water intoxication and hyponatremia, which makes it unsuitable for patients with polydipsia (26). DDAVP has a varying success rate in treating enuretic children. About one-third of patients experience significant improvement; another third reports no change, and the remaining third shows moderate results. However, relapses can occur in up to 70% of cases when the medication is interrupted (25), particularly without structured desmopressin withdrawal, which could help to reduce the risk of relapse (27).

It is estimated that approximately one-third of patients with MNE may require additional treatment after first-line interventions. This can be a challenging situation for pediatricians and urologists (28).

Parasacral transcutaneous electrical nerve stimulation (PTENS) is a treatment modality widely used to manage lower urinary tract dysfunction (LUTD), that failed first-line conservative therapies (29-31). Its mechanism of action in LUTD still needs to be clarified. It is believed that it reorganizes the action or expression of impulses and inhibitory impulses (neurotransmitters or receptors) in the bladder to reverse or recover the organ’s function (32, 33). Electric current through the hypogastric nerve activates inhibitory sympathetic neurons and inhibits excitatory parasympathetic neurons (pelvic nerve), promoting central nervous system reorganization and preventing involuntary detrusor muscle contractions (34). Stimulating the sensory afferents S2 and S3 can help regulate involuntary bladder contractions by inhibiting the pontine micturition center. This interruption of excessive detrusor stimulation leads to the restoration of normal micturition reflex. This process, also, causes sensory accommodation by reducing the excitability of ascending sympathetic nerves. (35). Theoretical research suggests that using electrical nerve stimulation during childhood may improve the central and peripheral nervous system’s neuroplasticity, leading to potentially better long-term outcomes (36). In addition to its established use in treating overactive bladder, recent randomized clinical trials have demonstrated varying effects of PTENS in treating MNE (29, 37-39). According to Bastos Netto et al. (6) PTENS could be tried in cases where other therapies have failed.

It is essential to understand that children and adolescents with enuresis often have low self-esteem and low quality of life. This medical condition can also negatively impact their academic performance and social life, especially if they are subjected to verbal or physical abuse by their caregivers. Enuresis is typically punished, highlighting the need to educate family members about its involuntary nature and the importance of treatment (40, 41). Thus, finding an effective treatment for children and adolescents with enuresis who do not respond to first-line therapies is crucial. Therefore, this systematic review aims to present the latest literature on the effectiveness of PTENS as a potential treatment option for MNE.

## METHODS

The Preferred Reporting Items for Systematic Review and Meta-Analyses (PRISMA) statement guided this systematic review (42) (Supplement 1). The review protocol was registered at the International Prospective Register of Systematic Reviews (PROSPERO), registration number CRD42021269279 (43).

### Eligibility criteria

The PICO (Problem or Population, Interventions, Comparison and Outcome or Result) structure was used in the development of the search for an answer to the main question of this review: *Is PTENS effective in treating children and adolescents with MNE?* The acronym PICO in this review stands for:

– P (population or problem): children and adolescents between six and 17 years who have been diagnosed with monosymptomatic enuresis according to ICCS criteria.
– I (intervention): transcutaneous electrical stimulation of the parasacral nerve.
– C (comparison): comparison among children and adolescents who received transcutaneous electrical stimulation of the parasacral nerve and those who did not.
– O (outcome or result): the efficacy of the treatment in reducing the number of wet nights. According to the ICCS criteria, treatment success is categorized into three groups: no-response (<50% symptom reduction), partial response (50 to 99%) and complete response (>100%) (1, 2).

Any articles that involved children or adolescents diagnosed with enuresis but who did not meet ICCS criteria, which used medications that alter the action of the detrusor muscle or external urethral sphincter, which suffered from untreated attention deficit hyperactivity disorder, daytime incontinence, intellectual disability, diabetes mellitus, sickle cell disease, spinal cord injury, spina bifida, radiculopathy, or with urological malformations were excluded from this review.

### Literature search strategy

A comprehensive bibliographic search was conducted until September 2023 using the following databases: MEDLINE (via PubMed), Web of Science, SCOPUS, Central Cochrane Library, and Physiotherapy Evidence Database (PEDro). The search terms used were "electrical stimulation", "monosymptomatic enuresis", "bedwetting", "children", and "adolescent". Only Randomized Controlled Trials (RCTs) with no date or language limits were included. Reference checking of selected articles was also conducted to identify additional studies.

### Data extraction and storage

Two reviewers independently (MFD and FCCM) examined titles and abstracts to select eligible studies and filter out duplicates. Afterward, they assessed the studies’ titles and summaries to determine the relevant articles. When the reviewers encountered disagreement, they retrieved the full text of the article. Controversies were reconsidered and discussed until a consensus was reached. If controversies persisted, a third reviewer (MMAV) was consulted to make the final inclusion decision. All three reviewers evaluated the full text of the articles that were included in the final selection. To organize the information, a data extraction table was used.

The following data were extracted: study identification (first author, year of publication and country); participants (age, gender, sample size); study design; electrical stimulation variables (therapy type, number of participants in each group, home based PTENS or not, follow up, treatment protocol: pulse frequency, pulse width, number of sessions, frequency and duration of sessions, location of electrode); exclusion of polyuria, refractory to other treatments, key findings, inclusion and exclusion criteria, underwent urotherapy and result evaluation criteria.

All eligible studies were cataloged in an online library system, and those that did not meet the inclusion criteria were excluded, and the reasons for exclusion were documented.

### Assessment of risk of bias of individual studies

The analysis of the studies’ risk of bias included in this review was carried out using the tools "Risk of Bias tool for randomized trials" (Rob 2.0) (44) and "Risk of Bias VISualization (RoBVIS) (45). RoB 2.0 addresses five specific domains: (1) randomization bias; (2) bias from deviations from intended interventions; (3) bias regarding lack of outcome data; (4) bias in outcome measurement; and (5) bias in outcome selection (44). Two reviewers (EC and EML) utilized the tool independently. Discrepancies were resolved through discussion, with a third author (FCCM) acting as a referee when necessary.

### Methodological quality

To assess the methodological quality of clinical trials was used the PEDro scale. It assesses 11 items related to the study internal validity (two to nine) and statistical reporting (10 and 11), except for the first one (eligibility criteria), which is not computed in the total score (46). Scores of this scale range from zero to ten: scores <four indicate poor methodology, between four and seven fair quality, and from seven to ten higher quality (47). The scale was initially applied by two independent reviewers (MFD and FCCM), and in case of any disagreements, a third reviewer (MMAV) was consulted.

## RESULTS

### Study Selection

**Figure 1:**
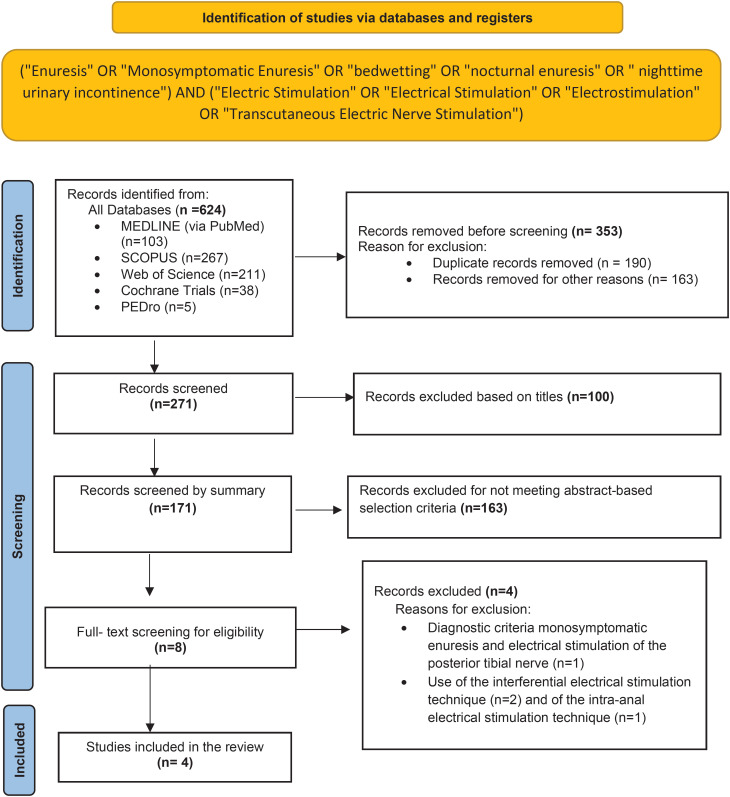
Flowchart with the research methodology following PRISMA guidelines.

A total of 624 studies were selected, with 103 results in MEDLINE (via PubMed), 267 in SCOPUS, 211 in Web of science, 38 in Central Cochrane Library and five in PEDro database. After the first screening, 353 were removed. Then, 100 were excluded based on title, 171 by the summary and eight were eligible to read the full text. One was excluded because of diagnostic criteria monosymptomatic enuresis and electrical stimulation of the posterior tibial nerve and three because other electro stimulation techniques were used. Figure-1 shows the flowchart summarizing the literature search process, following the PRISMA statement (42).

### Studies and participants characteristics

This review involved a total of 146 participants with MNE, ranging in age from six to 16.3 years. The intervention group (PTENS) had 92 participants, while the control group (CG) had 54 participants. One study did not have a control group and compared a group of 26 participants using PTENS to another group of 26 participants using transcutaneous interferential electrical stimulation, aged between seven and 14 years (39). Two studies did not have a placebo (37, 39). No statistical difference was found between genders. All studies were published between 2013 and 2023 and were randomized controlled trials (RCTs) (37-39, 48). These studies and participants’ characteristics are shown in Table-1.

**Table 1:** Sociodemographic characteristics of the included studies.

Study	Country	N	Male %	Age years Median interquartile range Mean ± SD	Study design
de Oliveira et al. 2013 (37)	Brazil	45	CG/CI: 50	CG: 9.9 ± 2.7 Range: 6.2-16.3 CI: 9.8 ± 2.9 Range: 6.3-14.1 (p=0.92)	RCT
Jorgensen et al. 2017 (48)	Denmark	47	CG: 74 IG: 88 (p=0.24)	CG: 9.1 ± 2.0 Range: 6-14 CI: 9.8 ± 2.2 Range: 6-14 (p=0.23)	RCT Double-Blind
Abdelhalim & Ibrahim, 2020 (39)	Egypt	52	PTENS G: 61.5 IFC G: 65.4 (p=0.77)	PTENS G: 10.9 ± 2.5 Range: 7-14 IFC G:10.3 ± 2.1 Range: 7-14 (p=0.35)	RCT single-blind
Oliveira et al. 2023 (38)	Brazil	28	CG: 33.3 IG: 37	CG: 8.76 ± 1.91 IG: 9,36 ± 2,52 (p=0.56) Not described median	RCT

SD = standard deviation; RCT = randomized controlled trial; IG = intervention group; CG = control group; PTENS = parasacral transcutaneous nerve stimulation; TIES = transcutaneous interferential electrical stimulation.

The study’s general characterization is presented in tables to help analyze their methodological quality. Table-2 provides details on the methods used in each study, including inclusion and exclusion criteria and the criteria used to evaluate treatment’s effectiveness. Table-3 shows the treatment protocol used by each author, including information on follow-up, PTENS parameters such as pulse duration and frequency, number and duration of sessions, electrode location, and main findings.

**Table 2:** Description of the methods of the included studies.

	Inclusion criteria	Exclusion criteria	Behavioral therapy	Criteria used for treatment evaluation	N Inicial/ final
de Oliveira, et al. 2013 (37)	Children older than 6 years diagnosed with primary MNE according to ICCS criteria	Children aged less than 6 years, presence of non-MNE or secondary enuresis, history of treatment for enuresis in the 6 months prior to study entry, and presence of urinary tract infection. neurological, psychiatric, or renal disease.	Yes	A decrease of less than 50% on wet nights determined no response, a decrease of 50% to 89% constituted a partial response, and a decrease of more than 89% indicated a response. To calculate the mean rate of improvement on wet nights for CG and IG, the formula %1/4 improvement [100 e (wet days after treatment/wet days before treatment)] was used.	45/38
Jorgensen et al. 2017 (48)	Children aged 6 to 14 years diagnosed with primary MNE, with a frequency of at least 3 nights per week and no treatment for nocturnal enuresis 1 week until the start of TENS treatment (2 weeks for enuresis alarm).	Children with nocturnal polyuria (defined as a nighttime urine volume greater than 130% of expected bladder capacity for age), ongoing constipation, and/or fecal incontinence; previous or continuous treatment with PTENS, presence of lower urinary tract infection, neurological or anatomical alterations of the urinary tract, or children undergoing operations on the urinary tract.	Yes	Non-response (less than 50% reduction in wet nights), partial responders (50% to 99% reduction) or complete responders (total dryness).	52/52
Abdelhalim & IbrahIm, 2020 (39)	Children and adolescents aged 7 to 14 years diagnosed MNE according to the ICCS with a history of three nights of nocturnal enuresis every week without any enuresis treatment for at least 1 month before the beginning of the study.	Children with neurological and/or psychological disorders, diagnosed with non-monosymptomatic or secondary enuresis, congenital anomalies, type I diabetes, urinary tract infection, and chronic constipation.	Yes	Non-response (0-49% wet nights reduced), partial response (50-89% wet nights reduced), good response (90% or more wet nights reduced), and full response (100% wet nights reduced); The percentage of progression % equal to [(number of wet nights pre-treatment) − (number of wet nights post-treatment)/ (number of wet nights pre-treatment] × 100	59/47
Oliveira et al. 2023 (38)	Children older than 5 years diagnosed with primary MNE and not being treated for enuresis or had any treatment in the past 6 months.	Families who showed no interest in participating in the study, those who had difficulty understanding the study objectives, patients with neurological, psychiatric, renal diseases, non-MNE and/or secondary enuresis.	Yes	Not described	72/45

MNE = monosymptomatic nocturnal enuresis; ICCS = International Children's Continence Society; TENS = transcutaneous electrical nerve stimulation; CG = control group; IG = intervention group.

**Table 3:** Descriptive summary of parasacral transcutaneous nerve stimulation data from included studies.

Study	Treatment protocol												
	Therapy in IG	Therapy in CG	IG (n)	CG (n)	Home-based PTENS	Follow-up	Pulse Frequency/Intensity	Pulse width (µs)	Number, frequency, and duration of sessions	Location of electrode	Exclusion of polyuric	Refractory to other treatments	Key findings
de Oliveira et al. 2013 (37)	PTENS	No placebo	27	18	No	6 months	10Hz	700	10 sessions 3/week 20 min	Sacral S2/S3	No	–	Improvement in MNE frequency was significant better in the EG (61.8%) when compared to CG (37.3%) No patient had complete resolution of symptoms. Combination of PTENS and behavioral therapy decreased more the percentage of wet nights compared to behavioral therapy alone.
Jorgensen et al. 2017 (48)	PTENS	Placebo	24	23	Yes	10 weeks	10Hz. Intensity was the highest tolerable level up to a maximum of 40mA.	200	140 home sessions 2/ day 60min	Sacral S2/S3	Yes	Yes (33)	No effect of PTENS treatment in participants without nocturnal polyuria, on MNE episodes, nocturnal urine production, or bladder reservoir function.
Abdelhalim & IbrahIm, 2020 (39)	PTENS & TIES	No placebo No CG	PTENS: 26 TIES: 26	–	No	6 months	PTENS: 10Hz, with current intensity slowly increased to tolerance. TIES: 4000Hz	700	18 sessions 3/week 20 min	PTENS: Sacral S2/S3 TIES: Bilaterally placed on the symphysis pubic skin and the two other electrodes were crossly placed on the contralateral ischial tuberosity skin area.	No	–	The MNE frequency episodes reduced significantly and had improvement of quality of life in both groups.
Oliveira et al. 2023 (38)	PTENS	Placebo	15	13	No	T1: after 20^th^ session T2: 15 days after treatment T3: 30 days after treatment T4: 60 days after treatment T5: 90 days after treatment	10Hz	700	20 sessions 3/week 20 min	Sacral S2/S3	No	–	No patient had complete resolution of symptoms. The IG had a progressive reduction in the number of dry nights over 90 days of follow-up, while the CG did not maintain the improvement. Combination of PTENS and urotherapy decreased more the percentage of wet nights compared to urotherapy alone.

IG = intervention group; CG = control group; PTENS = parasacral transcutaneous nerve stimulation; TIES = transcutaneous interferential electrical stimulation.

### Protocol and procedures

The studies used similar PTENS protocols. All of them used the same electrode location (S2/S3 sacral region) and the same frequency (10Hz) (37-39,48). Three studies used the same pulse duration (700µs), therapy time (20 minutes), and therapy frequency (three times a week) (37-39). Only Jorgensen et al. (48) used home-based PTENS with a pulse duration of 200µs, 60 minutes of application per day and a therapy frequency of twice a day. The total number of sessions varied between ten and 140, and the follow-up between 90 days and six months (Table-2).

Two studies used a minimum age of six years as an inclusion criterion (38, 48), one of five years (37) and the others, of seven years (39). All studies excluded patients with non-monosymptomatic enuresis, secondary enuresis, and neurological disease (37-39, 48). One study also excluded a patient with polyuria (48). More details are described in Table-2.

### Risk of bias assessment

**Figure 2:**
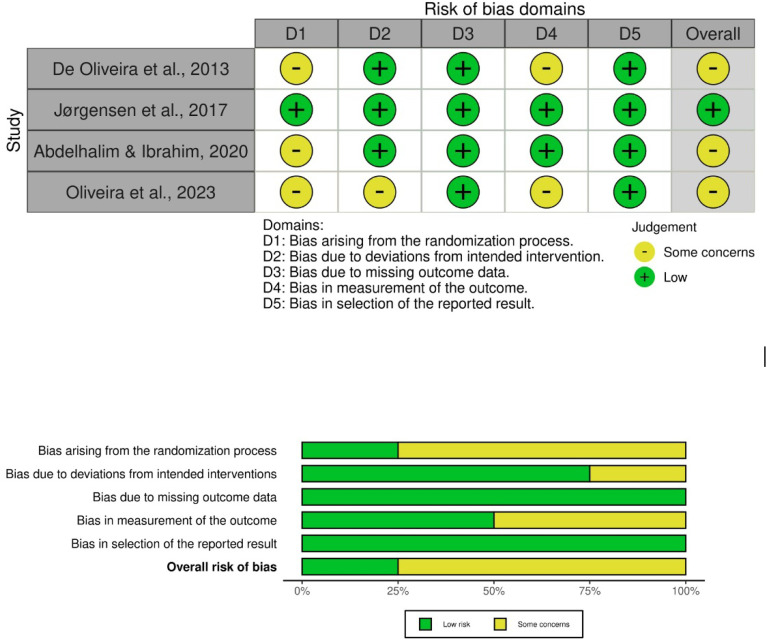
Summary and graph of the risk of bias for the included studies based on the Risk of Bias tool for randomized trials -ROB 2.0.

The risk of bias summary of the four included studies is shown in Figure-2. One study showed low risk of bias in all domains (48), and the other three showed some concerns, mainly arising from randomization and outcome measurement (37-39).

### Methodological quality

The evaluation of methodology’s quality is explained in detail in Table-4. Two studies received a score of five on the PEDro scale (37, 38) and one a score of seven (39), showing reasonable quality indicating a limited level of evidence regarding the benefits of PTENS in enuresis. Another study was classified as higher quality, with a score of ten (48). The most common methodological flaws were the absence of allocation concealment, blinding of patients, therapists and evaluators, and intention-to-treat analysis.

**Table 4:** Quality analysis of studies included in the Physiotherapy Evidence Database (PEDro) Scale.

PEDro scale	de Oliveira et al. 2013 (37)	Jorgensen et al. 2017 (48)	Abdelhalim & Ibrahim, 2020 (39)	Oliveira et al. 2023 (38)
1. Eligibility criteria were specified.	Yes	Yes	Yes	Yes
2. Patients were randomly allocated to groups (in a crossover study, they were randomly allocated to groups according to the treatments received)	Yes	Yes	Yes	Yes
3. Allocation concealed	No	Yes	Yes	No
4. Groups were similar at baseline regarding the most important prognostic indicators	Yes	Yes	No	Yes
5. Blinding of all patients	No	Yes	No	No
6. Blinding of all therapists who administered the therapy	No	Yes	No	No
7. Blinding of all assessors who measured at least one key outcome	No	Yes	Yes	No
8. Measures of at least one key outcome obtained from more >85% of the patients initially allocated to groups	Yes	Yes	Yes	Yes
9. All patients for whom outcome measures were available received treatment or sham as allocated or, where this was not the case, data for at least one key outcome were subjected to intention-to-treat analysis	No	Yes	Yes	No
10. Results of between-group statistical comparisons were reported for at least one key outcome	Yes	Yes	Yes	Yes
11. Study provided both point measures and measures of variability for at least one key outcome	Yes	Yes	Yes	Yes
**Total score**	**5**	**10**	**7**	**5**

### Main findings

This review assessed the efficacy of PTENS in MNE based on the number of wet nights, according to ICCS criteria, in four selected RCTs (37-39,48). A single study described a complete response to treatment in 27% and 19.8% of patients immediately and six months after the last session, respectively (39).

In a study conducted by de Oliveira et al. (37), children and adolescents with MNE were randomly assigned to two groups. The first group was treated with a standard urotherapy, while the second group was treated with both standard urotherapy and PTENS. The results showed that the second group (IG) had a significant improvement in wet nights of 61.8%, while the first group (CG) only had an improvement of 37.3% (p=0.003). However, no patient in either group achieved complete improvement.

Jorgensen et al. (48) compared children randomized in two groups, both were treated with a standard urotherapy. The IG received home-based PTENS therapy, while the CG received sham home-based PTENS therapy. However, there was no reduction in the number of wet nights, nocturnal urine production or bladder reservoir function characteristics.

In a study conducted by Abdelhalim & Ibrahim (39), two different modes of treatment were compared - PTENS and transcutaneous interferential electrical stimulation. The study found that both modes of treatment led to a significant decrease in the number of wet nights and an improvement in the participants’ quality of life. However, the group that received transcutaneous interferential electrical stimulation showed greater immediate and short-term improvements sustained over a more extended period than the PTENS group (p <0.05).

According to the latest study conducted by Oliveira et al. (38), both the CG and IG were given standard urotherapy. However, the IG received an additional treatment of PTENS, while the CG received a placebo version of PTENS. Four evaluations were carried out after the intervention, the first, immediately, after the intervention and the last 90 days later. There was a progressive improvement in the number of dry nights in each evaluation, with a significant improvement in IG comparing the pre-treatment values with the values found after 90 days (p<0.00).

## DISCUSSION

This review aimed to answer whether the PTENS is effective in treating MNE in children and adolescents. Although there are few randomized clinical trials available, this review found that PTENS can effectively reduce the number of wet nights per week, but in most cases, it only shows a partial response. The included studies are heterogeneous, making them unsuitable for meta-analysis. Despite the differences, in all the studies, the children in both groups received urotherapy and used the same electrostimulation frequency parameter (10Hz) (37-39). Only one of the articles used different methods, and the therapy was conducted at home (48). Interestingly, this was the only study that found no improvement in any of the outcomes assessed. This outcome prompts us to consider two reflections. Firstly, the significance of the parameters that were used, and secondly, the quality of the technique that was employed.

PTENS has shown promising results in treating other types of LUTD, such as overactive bladder and non-monosymptomatic enuresis (29, 30, 31). Although there is no agreed-upon definition of the optimal parameters to be used, most of the studies utilize the same parameters as those used by de Oliveira et al. (37), Abdelhalim & Ibrahim (39), and Oliveira et al. (38). Furthermore, the technical quality of the intervention carried out by a trained professional is undoubtedly better when compared to the technique of a lay person. In this case, appropriate positioning of electrodes and adjusting the current amplitude to the maximum sensory threshold can improve the therapeutic response. However, Jϕrgensen et al. (48) did not fully address these two requirements in the study.

Although the exact PTENS’ mechanism of action is still unclear, a study conducted by Netto et al. (49) has shown increased connectivity between the anterior cingulate cortex and the dorsolateral prefrontal cortex. This leads to a balance of sympathetic and parasympathetic stimuli in the bladder, promoting central nervous system reorganization and preventing involuntary detrusor muscle contractions, as demonstrated by Lindstrom et al. (50). Considering that one of the tripods in the pathophysiology of enuresis is detrusor hyperactivity, it is expected that PTENS will have a positive effect on at least this causal factor. In a recent meta-analysis, several treatments for overactive bladder in children were compared for their effectiveness and safety. The study found that PTENS was the best therapeutic option for improving maximum urinary volume, followed by urotherapy when compared to antimuscarinics. (51) It is known that as bladder capacity increases, it becomes possible to store more urine, which may be enough to accommodate urine production during sleep without the need to urinate.

It is still being determined whether standard urotherapy should be the first line of treatment for MNE (52). However, it is established that it can be used to treat other types of LUTD. Its effectiveness seems to be related to the greater intensity of treatment (53). In this review, studies that performed PTENS in person had a greater opportunity to reinforce standard urotherapy with frequent contact with the professional, which could be considered a confounding factor.

Two studies have demonstrated that symptoms gradually improve over the course of treatment (37, 38). The first study, conducted by de Oliveira et al. (37), involved only ten sessions, while the second study (38) involved 20 sessions. Both studies found that patients responded positively to treatment. We were unable to find any other clinical trial that investigated the relationship between the number of sessions and the gradual improvement of MNE. However, Veiga et al. (54) conducted a survey to evaluate the effectiveness of PTENS in overactive bladder per session. They observed that improvement occurred gradually, with more significant improvement after the 13th session. The improvement curve continued to increase until the end of the treatment at the 20th session. It was suggested that if treatment continued beyond the 20th session, an increased number of patients would have showed improvement.

A recent systematic review showed that using PTENS to treat enuresis is of no benefit. However, it is worth highlighting that this review included studies with patients who had non-monosymptomatic enuresis (55). In contrast, our review focused only on patients with monosymptomatic enuresis and found that PTENS can effectively reduce the number of wet nights. However, only one of the RTCs (39) showed complete remission in 27% of patients. We believe that the effectiveness of the treatment depends on the parameters used and the correct treatment technique applied by the professional. Effective treatment of enuresis is crucial due to its prevalence and impact on the quality of life of children and their families. (4, 40, 56). Although first-line therapies are well established, they are often ineffective and have high discontinuation rates (4, 24, 25). Therefore, it is essential to have robust studies that prove the effectiveness of alternative treatments such as PTENS.

It’s worth noting that there are certain limitations to this review. Firstly, we only included a few studies due to our strict inclusion criteria, which involved complying with the ICCS guidelines and selecting only randomized controlled trials. Another limitation was the relatively small sample sizes of the studies. Additionally, due to the heterogeneous nature of the data, it was not possible to conduct a meta-analysis.

## CONCLUSION

According to the review, PTENS can reduce the occurrence of wet nights in children and adolescents with MNE. However, it is not a complete cure for the condition, except for one study that reported a 27% cure rate among patients. To determine the most effective protocol for this treatment, more high-quality research is needed. Comprehensive evaluation of its effectiveness will require larger samples and more sessions.

## ABBREVIATIONS

BVS: = Biblioteca virtual em saúde
CG: = Control group
DDAVP: = Desmopressin acetate
ICCS: = International Children´s Continence Society
IG: = Intervention group
LUTD: = Lower urinary tract dysfunction
MNE: = Monosymptomatic enuresis
PEDro: = Physiotherapy Evidence Database
PICO: = Problem or Population, Interventions, Comparison and Outcome
PRISMA: = Preferred Reporting Items for Systematic
PROSPERO: = International Prospective Register of Systematic Reviews and Meta-Analyses
PTENS: = Parasacral transcutaneous electrical nerve stimulation
ROB2: = Risk-of-bias in randomized trials
ROBVIS: = Risk-of-bias visualization
RTCs: = Randomized controlled trials
TIES: = Transcutaneous interferential electrical stimulation
